# Nocardiosis - an emerging complication in the clinical management of HIV infected patients

**DOI:** 10.1186/1742-4690-9-S1-P134

**Published:** 2012-05-25

**Authors:** Chioma Onyinye Nwuba, Gabriel Kogo, Ngozi Ogbu, Oluwafemi Abolarin, Robert Okonkwo

**Affiliations:** 1 Pro-Act, Msh, Ilorin, Nigeria

## Introduction

In Nigeria as well as other parts of Africa, little is known regarding the prevalence of nocardiosis among HIV positive patients. Nocardiosis is usually not considered in the differential diagnosis for tuberculosis (TB) since it is not regarded as an AIDS defining illness.

The aim of this study is to determine the prevalence of nocardiosis in HIV positive patients suspected of having tuberculosis.

## Materials and methods

In this prospective study, sputum samples of 234 HIV positive patients with suspected cases of pulmonary tuberculosis were analyzed for Mycobacterium TB and Nocardia specie. Each sample was processed by conventional Ziehl Neelsen stain and examined for the presence of acid fast bacilli (AFB). AFB negative samples were streaked on slopes of Sabouraud’s dextrose agar and paraffin coated glass rods which acted as bait for Nocardia was introduced into each of these inoculated media and incubated at 37°C. Cultures were examined after two weeks for the presence of cream/orange tufts around the rods suggestive of nocardia. The isolates were scraped and further identified using biochemical tests. The CD4 cell count of each patient was estimated using Becton Dickenson FACS count system.

## Results

Of the 234 patient samples examined, 8 had positive culture for Nocardia. The prevalence of TB was 10.3% while that of Nocardia spp was 3.4%. All cases of nocardiosis detected was found in patients with CD4 count of <200cells/ul with 75% of these cases having CD4 count below 100cells/ul.

Out of the 8 patients diagnosed with nocardiosis in this study, 7 (7.4%) were not receiving antiretroviral therapy (ART) while 1(0.7%) with a CD4 count of 109 cells/ul had already commenced ART. We also observed that all 8 patients diagnosed with nocardiosis all had a negative AFB result after producing three sputum samples for TB analysis.

## Conclusions

In Nigeria where HIV-related tuberculosis occurs frequently, some patients diagnosed as having sputum smear-negative pulmonary tuberculosis actually have nocardiosis. It is pertinent that TB laboratories include gram staining during routine investigations of sputum samples especially for patients who present with typical features of active tuberculosis but whose smears are repeatedly negative for AFB.

